# Double Deletion of PI3K and PTEN Modifies Lens Postnatal Growth and Homeostasis

**DOI:** 10.3390/cells11172708

**Published:** 2022-08-30

**Authors:** Caterina Sellitto, Leping Li, Thomas W. White

**Affiliations:** Department of Physiology & Biophysics, Stony Brook University School of Medicine, Stony Brook, NY 11794, USA

**Keywords:** mouse, lens, knockout, PTEN, PI3K, Akt

## Abstract

We have previously shown that the conditional deletion of either the p110α catalytic subunit of phosphatidylinositol 3-kinase (PI3K), or its opposing phosphatase, phosphatase and tensin homolog (PTEN), had distinct effects on lens growth and homeostasis. The deletion of p110α reduced the levels of phosphorylated Akt and equatorial epithelial cell proliferation, and resulted in smaller transparent lenses in adult mice. The deletion of PTEN increased levels of phosphorylated Akt, altered lens sodium transport, and caused lens rupture and cataract. Here, we have generated conditional p110α/PTEN double-knockout mice, and evaluated epithelial cell proliferation and lens homeostasis. The double deletion of p110α and PTEN rescued the defect in lens size seen after the single knockout of p110α, but accelerated the lens rupture phenotype seen in PTEN single-knockout mice. Levels of phosphorylated Akt in double-knockout lenses were significantly higher than in wild-type lenses, but not as elevated as those reported for PTEN single-knockout lenses. These results showed that the double deletion of the p110α catalytic subunit of PI3K and its opposing phosphatase, PTEN, exacerbated the rupture defect seen in the single PTEN knockout and alleviated the growth defect observed in the single p110α knockout. Thus, the integrity of the PI3K signaling pathway was absolutely essential for proper lens homeostasis, but not for lens growth.

## 1. Introduction

Class I phosphoinositide 3-kinases (PI3Ks) are lipid kinases that phosphorylate the 3′-hydroxyl group of phosphatidylinositol-(4,5) P_2_ following the activation of cell surface receptors. In turn, the phosphatidylinositol-(3,4,5)P_3_ (PIP_3_) generated goes on to activate additional signaling pathways that regulate cell growth, proliferation, motility, and survival [1]. Class IA PI3Ks are heterodimers composed of an 85 kD regulatory subunit and a 110 kD catalytic subunit. The p110α catalytic subunit is widely expressed, and responds to input from receptor tyrosine kinases [2]. PI3K generates PIP_3_, which leads to the activation of the Akt signaling pathway [3,4,5,6]. Mutations in the gene encoding p110α are often linked to cancer, highlighting the importance of the PI3K signaling cascade in cell proliferation and survival [1]. Phosphatase and tensin homolog (PTEN) is a widely expressed lipid phosphatase that antagonizes PI3K signaling [7,8]. PTEN dephosphorylates PIP_3_ generated by PI3K and negatively regulates Akt activity [3,9]. The interplay between PTEN, PI3K, and Akt controls numerous cellular processes in many organs, including the ocular lens [10,11,12,13,14].

The anterior surface of the lens is covered by a monolayer of epithelial cells, which differentiate into fiber cells at the equator and fill the lens core [15]. Epithelial cell proliferation drives the growth of the lens, and primarily occurs in a band of cells near the equator called the germinative zone [16,17,18]. Lens development and growth are influenced by growth factor signaling. For example, fibroblast growth factors (FGFs) and fibroblast growth factor receptors (FGFRs) regulate lens induction, epithelial cell proliferation, and fiber differentiation [19,20,21]. Once activated by FGFs, FGFRs stimulate the mitogen-activated protein kinase (MAPK) and PI3K–Akt intracellular signaling pathways [10,22,23]. Genetic dissection experiments have illuminated the roles of many components of the MAPK pathway in the lens [24,25,26,27,28,29,30]. In contrast, the roles played by PI3K, Akt, and PTEN in these processes are less well characterized. One recent study examined the effects of the lens-specific knockout of p110α [13]. The loss of p110α substantially reduced phosphorylated Akt levels and caused a reduction in the number of proliferating lens epithelial cells present in the germinative zone on postnatal day 0 (P0), resulting in significantly reduced lens size throughout life. Two additional studies reported the consequences of the lens-specific deletion of PTEN, the lipid phosphatase that antagonizes PI3K [7,8]. In both studies, the levels of phosphorylated Akt were significantly elevated following the loss of PTEN, with different consequences related to the developmental timing of lens-specific knockout [14,31]. The loss of PTEN at the lens placode stage rescued a cell death phenotype caused by the knockout of the FGFR2 [31]. The knockout of PTEN at the lens vesicle stage reduced Na^+^/K^+^-ATPase activity, which caused the intracellular hydrostatic pressure to increase, leading to lens swelling and rupture [14].

Here, we have generated and characterized a conditional double-knockout mouse lacking both p110α and PTEN in the lens using a Cre recombinase active at the lens vesicle stage [32]. We evaluated the epithelial cell proliferation on P0, levels of phosphorylated Akt, lens growth, and lens homeostasis. We found that the double deletion of p110α and PTEN rescued the deficits in germinative zone proliferation and lens growth seen after the single knockout of p110α [13], but exacerbated the lens rupture phenotype seen in PTEN single-knockout mice [14]. The levels of phosphorylated Akt in double-knockout lenses were elevated 4.5-fold compared to wild-type lenses. These results document that the double deletion of p110α and PTEN alleviated the proliferation and growth deficits, and exacerbated the lens rupture defects seen in the respective single knockouts.

## 2. Materials and Methods

### 2.1. Generation of Knockout Mice

All animal use was approved by the Stony Brook University Institutional Animal Care and Use Committee. Mice with lens-specific conditional knockouts of the p110α catalytic subunit of PI3K [13], or PTEN [14], were interbred to obtain double-knockout animals (p110α/PTEN dKO). Briefly, p110α homozygous floxed [33] and PTEN homozygous floxed mice [34] were interbred with heterozygous MLR10-Cre mice [32]. Genotypes were verified by a PCR of tail DNA as previously described [13,14]. Due to the mixed genetic background that resulted from the interbreeding of three different strains of original mice, littermate controls were used for all experiments. All animals used for breeding had homozygous floxed alleles for both PTEN and p110α, while one member of the breeding pair carried a heterozygous copy of the MLR10-Cre transgene. The resulting litters contained one half of the animals with no Cre (wild-type controls) and one half with the double knockout of PTEN and p110α (dKOs). No backcrossing of animals was performed. Some authors have reported spurious Cre-mediated lens and eye phenotypes that were not dependent on the deleted gene of interest [35]. We have twice published studies using the MLR10-Cre mice where no lens phenotype was observed following the successful knockout of floxed alleles for both Cx43 [36] and the p110β catalytic subunit of PI3K [13], and we feel that this is not an issue in the present study. No differences in phenotype were observed between male and female knockout mice.

### 2.2. Lens Growth Analysis and Photography

Littermate mice from birth to 24 weeks of age were euthanized by CO_2_ inhalation. Their eyes were removed and transferred to a tissue culture dish filled with 37 °C Tyrode solution on a prewarmed stage. Lenses were surgically removed and photographed using a SZX16 dissecting microscope attached to a digital camera (Olympus, Waltham, MA, USA). The diameters of lenses were measured from images and lens volume was calculated assuming a spherical shape. The incidence of lens rupture was also recorded [13,14,37].

### 2.3. Western Blotting

Lenses were surgically removed from dissected eyes and placed in Tyrode solution. Lens capsules were separated from the fiber cells of the lens using fine forceps, heat denatured in 2 × sample buffer, and then electrophoretically separated on SDS–PAGE gels. After being transferred to nitrocellulose membranes, western blots were probed with antibodies against PI3K p110α (rabbit monoclonal, Cell Signaling Technology, Danvers, MA, USA), antibodies against PTEN (rabbit monoclonal, Cell Signaling Technology, Danvers, MA, USA), antibodies against serine 473 phospho-Akt (mouse monoclonal, Cell Signaling Technology, Danvers, MA, USA), or antibodies against total AKT1/2/3 (rabbit polyclonal, Santa Cruz Biotechnology, Dallas, TX, USA). Peroxidase-conjugated goat anti-rabbit (Jackson ImmunoResearch, West Grove, PA, USA), or sheep anti-mouse (GE Healthcare, Pittsburgh, PA, USA) secondary antibodies were used to facilitate ECL detection. NIH ImageJ software (version 1.49v, http://imagej.nih.gov, USA, accessed on 22 August 2022) [38] was used to determine band densities from three independent blots.

### 2.4. 5-Ethynyl-2′-deoxyuridine (EdU) Staining

Postnatal day 0 (P0) mice were subcutaneously injected with 50 µg/gm EdU (Click-iT, Thermo Fisher Scientific, Waltham, MA, USA) and returned to their mothers for 2 h. Then, lenses were dissected and fixed in a 4% formaldehyde in PBS for 1 h and photographed. The permeabilization of fixed lenses and Click-iT staining were performed according to manufacturer’s instructions and previously published protocols [13,39]. Z-stacks of fluorescent images were acquired on an Axiovert 200 M microscope (Zeiss, Thornwood, NY, USA) and processed using ImageJ (version 1.49v, http://imagej.nih.gov, USA, accessed on 22 August 2022). For line-scan analysis, the flattened fluorescent image of EdU staining was manually thresholded in ImageJ (version 1.49v, http://imagej.nih.gov, USA, accessed on 22 August 2022), and the plot profile function was used in ImageJ to measure fluorescent intensity across the lens diameter as previously described [13].

### 2.5. Histology

Eyes from postnatal day 7 mice were dissected and fixed in 4% formaldehyde in phosphate-buffered saline (PBS) for 18 to 22 h at room temperature. After fixation, eyes were rinsed with PBS, dehydrated through a series of increasing ethanol concentrations and then embedded in paraffin. 2–3 µm sections were cut using a diamond knife and deparaffinized, before being stained with hematoxylin–eosin as previously described [13,14]. Histological sections were observed on a BX51 microscope and photographed with a DP72 digital camera (Olympus, Waltham, MA, USA).

### 2.6. Statistics

Data are presented as the mean ± SD, or SEM, as described in each figure. Statistical significance was determined using one-way ANOVA, or the two sample *t*-test in the Origin 2020 (64-bit) SR1software program (OriginLab Corporation, Northampton, MA, USA). P values that were less than 0.05 were considered significant.

## 3. Results

### 3.1. Lens-Specific Double Deletion of p110α and PTEN Caused Accelerated Cataract and Lens Rupture

**Figure 1:**
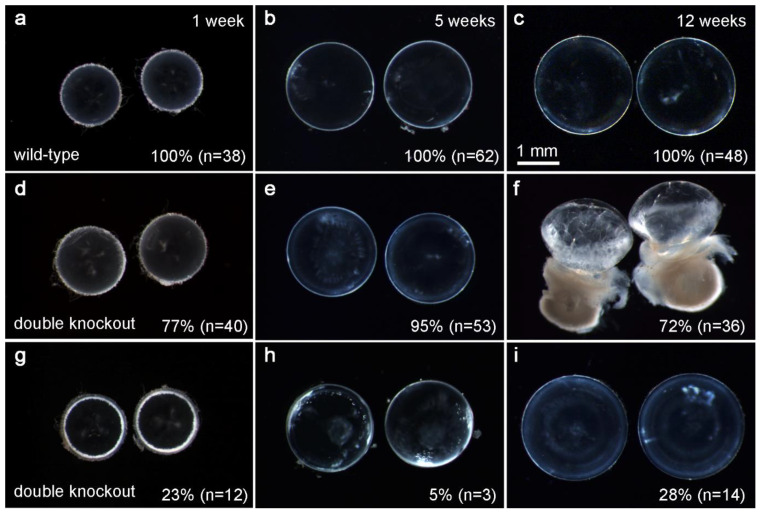
p110α/PTEN dKO mice develop cataract and lens rupture. Lenses dissected from wild-type (**a**–**c**) and p110α/PTEN double-knockout (**d**–**i**) mice are shown at 1, 5, and 12 weeks of age. Wild-type lenses (**a**–**c**) were transparent and intact at all ages studied. dKO lenses displayed a phenotype similar to single PTEN KO lenses [14], but with an altered time course. At 1 week, the majority of dKO lenses (**d**) appeared clear and of normal size when compared to WT (**a**). Only 23% of dKO lenses showed a ring of cortical vacuoles (**g**) as previously reported for 80% of single PTEN KO lenses at this age. At 5 weeks, only 5% of dKO lenses showed dispersed vacuoles (**h**), compared to 71% of single PTEN KO lenses at this age. At 12 weeks, 72% of dKO lenses had ruptured (**f**), compared to only 9% rupture for single PTEN KOs. The dense opaque material in panel f is the cataractous lens core. At 1 and 5 weeks, dKO lenses were of normal size (**d**,**e**,**g**,**h**), in contrast to single p110α KO lenses [13] which were significantly smaller than wild-type at all postnatal ages. n = number of lenses.

Mice with homozygous floxed p110α and PTEN alleles [33,34] were interbred with MLR10-Cre transgenic mice [32] to obtain lens-specific conditional double-knockout animals. MLR10-Cre mice express Cre recombinase in both the lens epithelium and fiber cells starting at the lens vesicle stage. Lenses isolated from homozygous floxed p110α/PTEN animals lacking the MLR10-Cre transgene (i.e., wild-type) remained transparent and intact at all ages examined (Figure 1a–c). In contrast, lenses dissected between 1 and 12 weeks of age from homozygous floxed p110α/PTEN animals expressing the MLR10-Cre transgene (i.e., p110α/PTEN dKO) showed a progressive pathology (Figure 1d–i). This phenotype was similar to that reported for the single PTEN KO lenses [14], but with an altered time course. At 1 week of age, the majority of p110α/PTEN dKO lenses (Figure 1d) appeared clear and of normal size. Only 23% of p110α/PTEN dKO lenses showed a ring of cortical vacuoles (Figure 1g) as previously reported for 80% of the single PTEN KO lenses at this age. At 5 weeks of age, only 5% of p110α/PTEN dKO lenses showed opacities dispersed throughout the outer lens cortex (Figure 1e,h), compared to 71% of single PTEN KO lenses at this age. By 12 weeks of age, 72% of p110α/PTEN dKO lenses had ruptured, leaving behind cortical fragments and a dense nuclear cataract (Figure 1f,i), compared to only 9% rupture for single PTEN KOs at this time point. Lenses that had not ruptured were noticeably larger than wild-type controls, and contained diffuse opacities. Thus, the double deletion of p110α and PTEN in the lens reduced the vacuole number, but accelerated the rupture phenotype described in single PTEN knockout mice [14].

### 3.2. Double Knockout of p110α and PTEN Increased Lens Volume and Accelerated Lens Rupture

**Figure 2:**
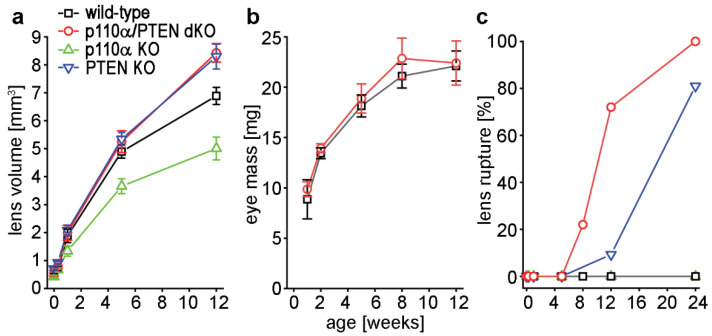
Double deletion of p110α/PTEN eliminated the growth defect of single p110α KO mice, and accelerated the rupture phenotype of single PTEN KO animals. (**a**) Diameters of lenses dissected from WT, p110α KO, PTEN KO and p110α/PTEN dKO mice at P0, P2, and 1, 5, or 12 weeks of age were used to calculate lens volume. The volumes of dKO lenses were similar to single PTEN KO lenses [14] at all ages. In contrast, the reduced lens volume reported for single p110α KO lenses [13] was absent. (**b**) Eye mass was not increased in dKO lenses when compared to WT. (**c**) The incidence of lens rupture versus age for WT, p110α KO, PTEN KO, and p110α/PTEN dKO mice at P0, P2, and 1, 5, 8, 12, and 24 weeks. Lens rupture was accelerated in dKO animals compared to single PTEN KO mice. No rupture was observed in wild-type or single p110α KO lenses. Data are plotted as mean ± SD. See Table S1 for a detailed breakdown of the number of lenses (4–64) analyzed per genotype per time point. See Table S2 for statistical comparison of dKO versus wild-type lens and eye growth per time point.

Changes in lens size and integrity were quantified over time by dissecting and photographing lenses from Postnatal day 0 (P0), P2, and 1, 5, and 12 week-old age-matched p110α/PTEN double homozygous floxed littermate animals, with or without the MLR10-Cre transgene. Single p110α and PTEN knockout lenses were also analyzed for comparison to the p110α/PTEN dKO animals. Lens diameters were measured and used to calculate volumes assuming spherical geometry [13,14,37]. As shown in Figure 2a, the effect of p110α/PTEN dKO on lens size developed slowly, with no significant differences in volume compared to wild-type observed between P0 and 1 week of age. In contrast, a 7% increase in lens volume in p110α/PTEN dKO lenses developed between 1 and 5 weeks when compared to wild-type (*p* < 0.05, Student’s *t*-test), which grew to a 22% volume increase by 12 weeks in the reduced number of intact dKO lenses still available at that age (*p* < 0.05). As previously reported [13,14], p110α single KO lenses were significantly smaller than wild-type at all postnatal ages studied, and PTEN single KO lenses showed a volume increase comparable to the p110α/PTEN dKOs. The overall eye size, as expressed by eye mass, was also similar between wild-type and p110α/PTEN dKOs animals (Figure 2b). This contrasted with the significantly decreased eye size previously reported for p110α single KO animals [13]. We further recorded the frequency of lens rupture between P0 and 24 weeks for wild-type and p110α/PTEN dKO littermate animals, and compared these data with the rupture frequency observed in single p110α and PTEN KOs. Wild-type, or single p110α KO, lenses never exhibited lens rupture in the time period examined, while 22% of p110α/PTEN dKO lenses had ruptured by 8 weeks, which increased to a 100% incidence of rupture at 24 weeks (Figure 2c). In contrast, only 9% of single PTEN KO lenses had ruptured by 12 weeks, and 19% of PTEN KO lenses were still intact at 24 weeks of age. These data suggested that although the activity of p110α alone was required by the lens to grow to a normal lens volume [13], further removal of PTEN in p110α/PTEN dKO lenses alleviated this growth deficit. They also showed that although the loss of PTEN alone resulted in a loss of lens ionic homeostasis and ultimate lens rupture [14], the further removal of p110α in p110α/PTEN dKO lenses greatly accelerated this loss of lens homeostasis.

### 3.3. Double Knockout of p110α and PTEN Increased Levels of Phospho-Akt

**Figure 3:**
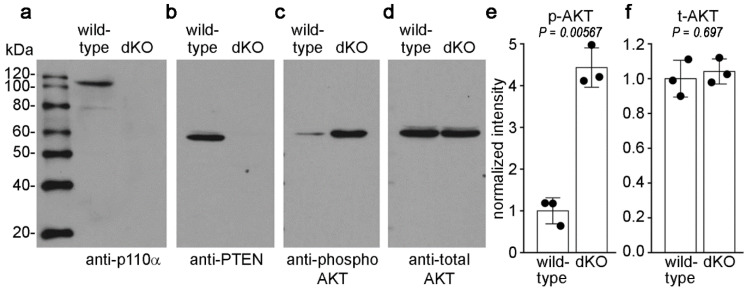
Double deletion of p110α/PTEN increased levels of phospho-AKT. Lens capsules with attached epithelial cells were isolated from 3 week old WT or dKO lenses, and used for western blots. Blots were probed with antibodies against p110α, PTEN, phospho, or total AKT. Both p110α (**a**) and PTEN (**b**) proteins failed to be detected in dKO lenses. (**c**) As previously reported for single PTEN KO lenses [14], phospho-AKT (p-AKT) levels were elevated in dKO lenses. (**d**) Total AKT (t-AKT) levels were the same in wild-type and dKO lenses. Quantitation of band intensities showed a ~4.5-fold increase in p-AKT levels in dKO lenses (**e**, *p* < 0.05, two sample *t*-test), and no significant differences in t-AKT levels between wild-type and dKO mice (**f**, *p* > 0.05 two sample *t*-test). Raw data are plotted as filled circles, bars represent the mean ± SD, n = 3.

To confirm the absence of p110α and PTEN proteins, and determine how downstream Akt signaling was effected in dKO lenses, epithelial cell extracts were probed by western blot (Figure 3a–d). Wild-type epithelial cells expressed both the p110α catalytic subunit of PI3K and its opposing phosphatase PTEN, which were completely absent from epithelial cells isolated from the dKO mice. Consistent with the presence of additional active PI3K isoforms in the lens [13,40] and PTEN’s antagonistic role toward PI3K signaling, the levels of phosphorylated Akt were elevated in p110α/PTEN dKO epithelial cells compared to wild-type. As expected, total Akt levels were not affected by p110α/PTEN double deletion. The quantitation of band densities (Figure 3e,f) showed no significant differences in levels of total Akt between wild-type and p110α/PTEN dKO epithelial cells (*p* > 0.05). In contrast, the levels of phospho-Akt were elevated approximately ~4.5-fold in p110α/PTEN dKO epithelial cells (*p* < 0.05). Full size images of replicate blots are shown in Figure S1. These data confirm that the loss of PTEN antagonism of PI3K activity resulted in elevated levels of phospho-Akt in p110α/PTEN dKO lenses, and further suggest that the absence of the growth defect observed in single p110α KOs may have been due to the restoration of elevated phospho-Akt levels in the dKO mice.

### 3.4. Double Knockout of p110α and PTEN Eliminated the Postnatal Proliferation Defect Present in Single p110α Knockout Lenses

**Figure 4:**
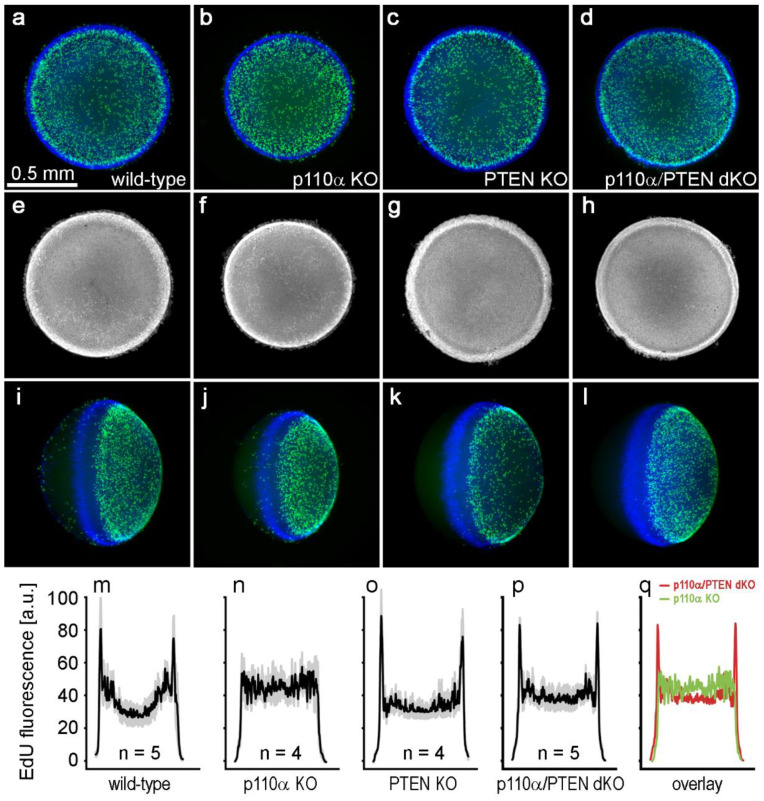
Double p110α/PTEN knockouts lacked the postnatal proliferation defect of single p110α knockout lenses. Postnatal day 0 lenses from wild type (**a**,**e**,**i**), single p110α KO (**b**,**f**,**j**), single PTEN KO (**c**,**g**,**k**), and p110α/PTEN dKO (**d**,**h**,**i**) mice were stained with EdU (green, **a**–**d**, **i**–**l**) and Hoechst (blue, **a**–**d**, **i**–**l**, or gray, **e**–**h**). Z-stack images were generated looking onto the epithelial surface (**a**–**d**), or onto the lens edge (**i**–**l**). Grayscale images of Hoechst-stained nuclei were also displayed to verify the overall integrity of the lens epithelium (**e**–**h**). EdU labeling showed that the distribution of proliferating cells in the p110α/PTEN dKO lenses was similar to that of wild-type, or single PTEN KO lenses, with the highest signal in the equatorial germinative zone. Plots of mean values of line scans (black lines, ± the SEM gray bars) of EdU fluorescence taken along the lens diameter (**m**–**q**) confirmed that the p110α/PTEN dKO lenses had recovered peak proliferation in the equatorial germinative zone, which was greatly reduced from single p110α KO lenses [13]. n = 4–5 lenses per genotype.

We have previously shown that the pattern of mitosis in p110α single KO lenses was significantly altered on P0, with the most prominent feature being a reduction in cell division in a band of cells known as the equatorial germinative zone of the lens epithelium [13]. To examine the effect of the double knockout of p110α/PTEN on postnatal mitosis, lenses from wild-type, single p110α and PTEN KO, or p110α/PTEN dKO mice were labeled with EdU on P0 (Figure 4). Wild-type, PTEN KO, and p110α/PTEN dKO lenses all displayed similar patterns of robust EdU labeling, with the highest level of fluorescence in the germinative zone near the lens equator as previously reported [13,41,42]. In contrast, single p110α KO lenses lacked the ring of increased labeling near the equatorial germinative zone, and showed a more homogeneous pattern of EdU incorporation as previously described [13]. The mean values of the line scans of fluorescent intensity taken along the lens diameter confirmed this change in mitotic pattern between EdU-labeled images from p110α KO and p110α/PTEN dKO lenses on P0. Wild-type, PTEN KO, and p110α/PTEN dKO lenses all had clear peaks of EdU fluorescent intensity near the equator. In contrast, p110α knockout lenses lacked the equatorial peaks, and had maximum values of fluorescence that ranged from 45% to 49% lower than either the wild-type, PTEN KO, or p110α/PTEN dKO lenses on average. Overlaying the average p110α KO trace onto that of the p110α/PTEN dKO lenses (Figure 4q) clearly showed that the lens equatorial proliferation peaks were recovered in the dKO animals.

**Figure 5:**
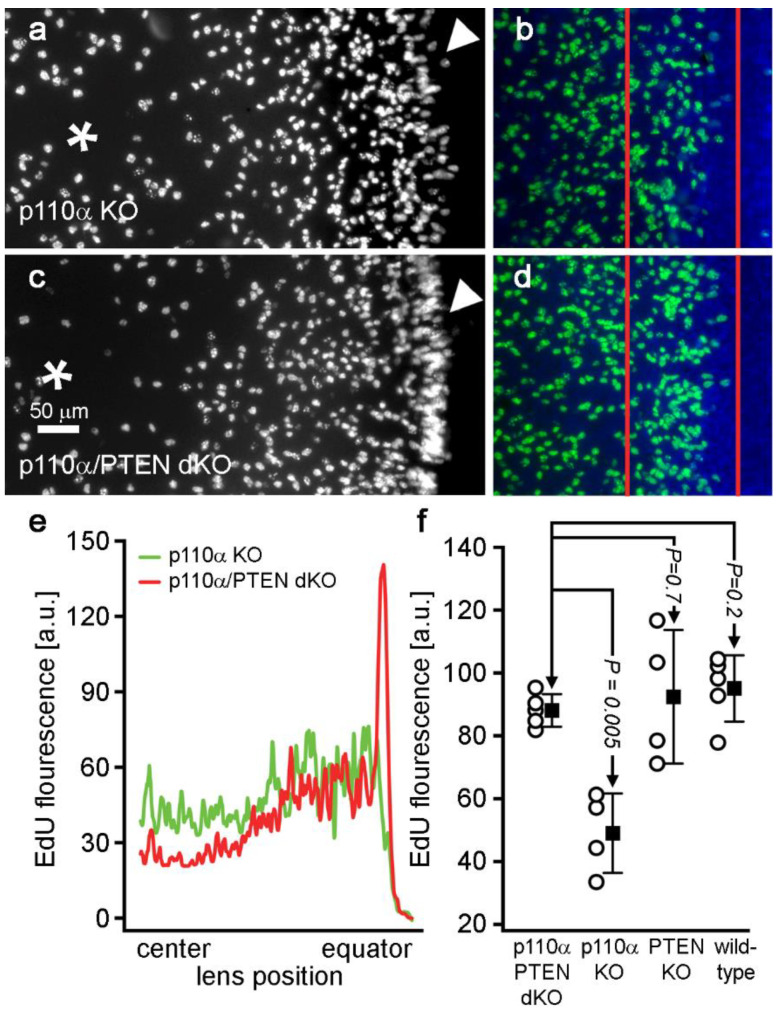
Germinative zone proliferation in double p110α/PTEN knockouts was significantly increased compared to single p110α knockout lenses. (**a**–**d**) Higher magnification views of postnatal day 0 lenses from single p110α KO (**a**,**b**) and p110α/PTEN dKO (**c**,**d**) looking onto the epithelial surface (**a**,**c**, EdU fluorescence alone), or onto the lens edge (**b**,**d**, EdU green, DAPI, blue). EdU-labeled cells are visibly increased in the germinative zone of p110α/PTEN dKOs in both views (white arrowheads in a and c, red lines in b and d). (**e**) Line scans of the entire field of view in panels a and c confirmed the increase in EdU fluorescence in the equatorial germinative zone in dKO lenses. (**f**) Plots of mean values of peak EdU fluorescence (open circles are raw data, filled squares are means ± the SD) showed a significant increase (*p* < 0.05) in p110α/PTEN dKOs compared to p110α KOs. There were no significant differences between p110α/PTEN dKOs and PTEN KO, or wild-type lenses (*p* > 0.5, two sample *t*-test). The center of the lens is indicated by an * in panels a and c. n = 4–5 lenses per genotype.

Higher power views of EdU fluorescence confirmed that equatorial germinative zone proliferation was restored in p110α/PTEN dKO lenses (Figure 5). A quantitative analysis of the peak EdU fluorescent signal detected in the germinative zone showed a 1.8-fold increase in p110α/PTEN dKO lenses compared to p110α single KOs (*p* < 0.05). There was no significant difference in peak EdU signal between the dKOs and PTEN single KOs, or wild-type lenses (*p* > 0.05). These data suggest that the significant reduction in lens volume, caused by a transient loss of proliferating epithelial cells in the germinative zone on P0 in single p110α KO lenses [13], was alleviated by the further removal of PTEN in p110α/PTEN dKO lenses.

### 3.5. Double Deletion of p110α/PTEN Produced Histological Abnormalities in the Equatorial Region of the Lens

**Figure 6:**
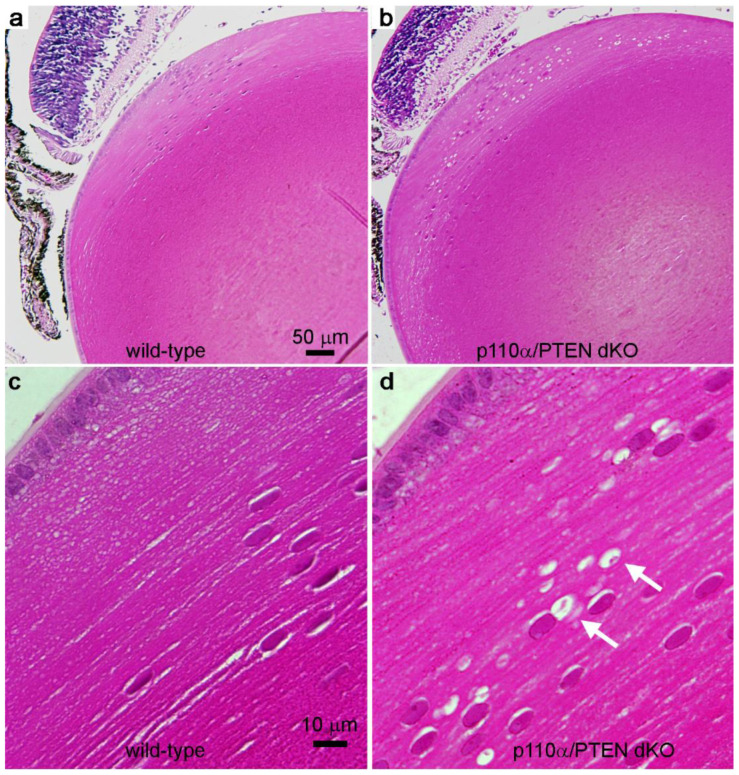
Postnatal day 7 lenses were fixed, embedded in paraffin, sectioned, and stained with hematoxylin and eosin. (**a**) Sagittal sections through the central region of wild-type lenses showed a normal appearance. (**b**) p110α/PTEN dKO lenses had numerous small vacuoles in the cortical region. (**c**) Higher power view of the cortex region of the wild-type lens shown in panel a. Higher power view of the cortex region of the p110α/PTEN dKO lens in panel b, showing the location of small vacuoles in the cortical fiber cells (white arrows). (**a**,**b**) Bar = 50 µm. (**c**,**d**) Bar = 10 µm.

To examine the histology of p110α/PTEN dKO lenses, we dissected eyes from littermate mouse pups on postnatal day 7, sectioned them, and stained with hematoxylin and eosin (Figure 6). Sagittal sections through the central region of wild-type lenses showed a normal appearance, whereas p110α/PTEN dKO lenses displayed small vacuoles in the equatorial cortex. Despite the presence of vacuoles, fiber cell differentiation appeared to proceed normally in dKO mice. Vacuoles have been reported in other mouse lens cataract models [14,26,27,43], and consistent with these reports, we observed an increase in the number of vacuoles in the dKO compared to wild-type. Histological abnormalities were not observed following the single knockout of p110α [13], unlike single PTEN KO lenses which showed more extensive vacuole formation at earlier ages than P7 [14]. Thus, the double deletion of p110α/PTEN from the lens resulted in milder histological defects in the lens cortex that appeared developmentally later than those observed in single PTEN KOs.

## 4. Discussion

We have generated p110α/PTEN double-knockout mice with the goal of improving our understanding of the integration of PI3K and PTEN signaling with the normal physiology and pathophysiology of the lens. The double deletion of p110α and PTEN produced a phenotype with features distinct from both of the respective single knockouts. The proliferation defect and smaller size of p110α KO lenses [13] was rescued by the further removal of PTEN in the dKO. In contrast, the rupture phenotype observed in PTEN KO lenses [14] remained, although the time course over which it developed was different. Vacuoles, which appeared as early as P2 in PTEN single Kos, were not visibly apparent before 1 week of age in the dKO. The rupture phenotype, on the other hand, was both accelerated and more penetrant. Phospho-Akt, which was reduced in p110α KO lenses, was elevated in both the PTEN KO and dKO.

Mouse lens growth undergoes a transition during the first postnatal week, the period where we previously reported a significant reduction in the number of dividing epithelial cells in p110α KO lenses [13]. Throughout embryonic development, the lens diameter grows linearly and its volume increases in an exponential manner [44,45]. Shortly after birth, the growth of the lens becomes oscillatory in a way corresponding to the epithelial cell cycle that can be reproduced in organ culture with the pulsatile administration of growth factors [46,47]. In the single p110α KO lenses, peak epithelial cell proliferation in the germinative zone transiently vanished at P0, despite being clearly present on E17 and P2 [13]. Lenses from p110α/PTEN dKOs maintained peak epithelial cell proliferation in the germinative zone at P0, and had elevated levels of phospho-Akt. These data show that the embryonic constant growth and the postnatal pulsatile growth mechanisms both produce maximum cell division in the germinative zone in the absence of p110α. However, the transition period between these mechanisms was sensitive to the loss of p110α activity alone, but not to the double deletion of p110a/PTEN. This suggests that elevated levels of phospho-Akt, whether generated through the activity of p110α, or the loss of PTEN, were required to maintain germinative zone proliferation during the transition between lens growth modes. The precise mechanism whereby Akt activity is required for the preservation of normal germinative zone proliferation around P0 would be a good subject for future studies.

The loss of the antagonism of PI3K signaling in single PTEN KO lenses increased the activation of Akt, and reduced Na^+^/K^+^-ATPase activity within the epithelial cells [14]. This caused a reduction in the normal sodium and water efflux mediated by the lens circulation [48,49], resulting in an increased intracellular hydrostatic pressure and ultimately lens rupture. The single PTEN lens KO phenotype initially displayed the formation of a ring of densely packed cortical vacuoles in neonatal mice that could be eliminated by in utero treatment with an Akt inhibitor [14]. Vacuole formation in the p110a/PTEN dKO was greatly reduced at both 1 week and 5 weeks of age, which was correlated with an overall reduction in the elevation of phospho-Akt levels in the p110a/PTEN dKO (4.5 times higher than wild-type control) compared to the PTEN KO (12 times higher than wild-type control). Thus, the reduced vacuole formation in the p110α/PTEN dKOs may have resulted from a failure to reach a sufficient threshold of Akt activation at the lens equator to disrupt cellular structure. Paradoxically, lens rupture in the p110α/PTEN dKO was greatly accelerated compared to the single PTEN KO, despite the fact that vacuole formation preceded lens rupture by several weeks. We have not directly measured the lens hydrostatic pressure in p110α/PTEN dKO lenses, and this would likely be difficult to achieve due to the high incidence of lens rupture. Since increased hydrostatic pressure is also known to be an Akt-dependent phenomenon [14,50,51], it is possible that differential levels of Akt activation are responsible for the accelerated rate of lens rupture in the p110α/PTEN dKO lenses. The elucidation of the precise signaling cascades that regulate lens hydrostatic pressure, including PI3K, PTEN, and Akt, will require further investigation.

Although this initial characterization of p110α/PTEN double-knockout lenses has elucidated several interesting findings, there remain a number of unanswered questions. Will the findings from this mouse model be relevant to human ocular health, or other vertebrate lenses? How are lens vacuoles initially produced, and why are they less abundant in the dKO than the PTEN single knockout? In addition to changes in phosphorylated Akt, what other downstream signaling molecules have their activity modulated by the combined loss of PI3K and PTEN? A better understanding of the relationship(s) between lens growth, the formation of lens vacuoles, changes in lens hydrostatic pressure, lens rupture, and the signaling cascades regulated by PI3K, Akt and PTEN will require additional experimentation.

We have examined PI3K/PTEN signaling in the lens using an in vivo methodology involving mice with multiple gene knockouts. PI3K/PTEN signaling critically regulates the activity of a broad network of lens ion channels and transporters, including connexins, TRP channels, and the Na^+^/K^+^-ATPase [14,50,51,52]. Future studies could examine mice with deletions of these transport proteins in conjunction with PI3K and/or PTEN knockout to further elucidate the mechanisms whereby this signaling cascade influences lens development and homeostasis. The systemic regulation of membrane channel/transporter activity by PI3K/PTEN has emerged as a novel homeostatic mechanism across many cell types and tissues. For example, the down regulation of PI3K signaling prolonged the QT interval of the cardiac action potential by affecting multiple ion channels, potentially explaining why some tyrosine kinase inhibitors in clinical use as anti-cancer therapies lead to an increased risk of arrhythmias, as with long QT syndrome [53,54]. In the central nervous system, the altered PTEN regulation of channels involved with synaptic transmission may explain abnormal social and cognitive behaviors observed in humans with PTEN hamartoma tumor syndrome (PHTS) and other neurological syndromes such as autism spectrum disorders [55,56]. In the initial segment of the epididymal epithelium, the targeted deletion of PTEN altered ion channel and transporter activity, resulting in reduced male fertility [57]. The loss of PTEN in thyroid cancer cells resulted in the altered plasma membrane expression of SLC2A1, and increased glucose uptake [58]. In a similar manner, PTEN has been linked to the altered activity of glucose transport, the ABCG2 transporter, and the glutamate aspartate transporter in other cell types [59,60,61].

Taken together, these observations suggest that PI3K/PTEN signaling may broadly participate in the regulation of the activity of diverse channels and transporters in many tissues, and that the loss of this regulation may contribute to a variety of developmental defects and disease states. Further study on how lens channels and PI3K/PTEN signaling work together to maintain clarity, preserve integrity, and regulate postnatal mitosis could provide broad insights into the regulation of channel/transport activity in many other tissues.

## Data Availability

The data presented in this study are contained within the article or supplementary material.

## Supplementary Materials

The following are available online at https://www.mdpi.com/article/10.3390/cells11172708/s1, Figure S1: Original Images for Blots, Table S1: Number of lenses analyzed per age and genotype for Figure 2. Table S2: Statistical comparison of lens and eye growth between wild-type and p110α/PTEN dKO mice.
