# Supplementary material for: Double Deletion of PI3K and PTEN Modifies Lens Postnatal Growth and Homeostasis
Source: Cells. 2022 Aug 30;11(17):2708. doi: 10.3390/cells11172708 (PMC9455000; doi:10.3390/cells11172708)

Original Images for Blots and Gels for the Article:

Double deletion of PI3K and PTEN modifies lens postnatal growth and homeostasis.

Caterina Sellitto, Leping Li and Thomas W. White

Full size images for the triplicate blots used to generate the images in Figure 3.

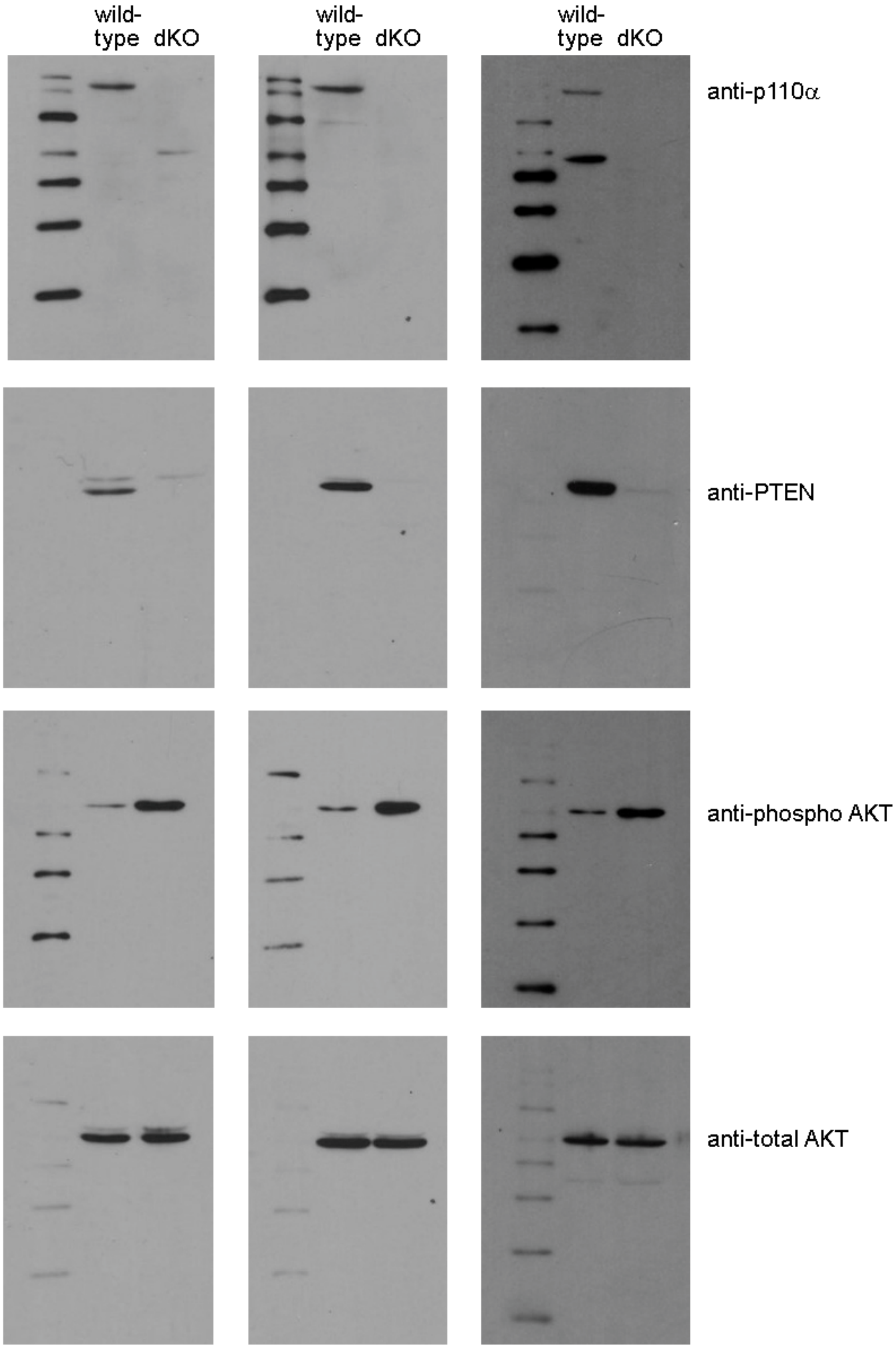

Supplement: Supplementary file 1 [file cells-11-02708-s001.zip › Figure S1 Original Images for Blots and Gels.pdf]
