# Supplementary material for: Double Deletion of PI3K and PTEN Modifies Lens Postnatal Growth and Homeostasis
Source: Cells. 2022 Aug 30;11(17):2708. doi: 10.3390/cells11172708 (PMC9455000; doi:10.3390/cells11172708)
Supplement: Supplementary file 1 [file cells-11-02708-s001.zip › Supplementary Table S1.pdf]

Supplementary Table S1. Number of lenses analyzed per age and genotype for Figure 2.

Figure 2, panels a and c				
Age	Wild-type	p110α KO	PTEN KO	p110α /PTEN dKO
P0	18	25	5	20
P2	7	37	9	4
1 week	38	52	30	52
5 weeks	62	59	52	56
8 weeks	22	0	0	18
12 weeks	48	64	39	50
24 weeks	58	63	46	64

Figure 2, panel b		
Age	Wild-type	p110α /PTEN dKO
1 week	20	24
2 weeks	16	6
5 weeks	36	26
8 weeks	22	18
12 weeks	42	42
