# Supplementary material for: Double Deletion of PI3K and PTEN Modifies Lens Postnatal Growth and Homeostasis
Source: Cells. 2022 Aug 30;11(17):2708. doi: 10.3390/cells11172708 (PMC9455000; doi:10.3390/cells11172708)
Supplement: Supplementary file 1 [file cells-11-02708-s001.zip › Supplementary Table S2.pdf]

Supplementary Table S2. Statistical comparison of lens and eye growth between wild-type and p110α/PTEN dKO mice from the data presented in Figure 2.

Lens growth			
Age	Wild-type lens volume [mm^3] (mean ± SD)	p110α/PTEN dKO lens volume [mm^3] (mean ± SD)	P value (two sample t-test)
P0	0.50 ± 0.04	0.55 ± 0.11	0.068
P2	0.70 ± 0.06	0.77 ± 0.06	0.106
1 week	1.88 ± 0.24	1.99 ± 0.27	0.041
5 weeks	4.89 ± 0.23	5.25 ± 0.39	1×10^{-8}
12 weeks	6.89 ± 0.30	8.43 ± 0.33	1×10^{-13}

Eye growth			
Age	Wild-type eye mass [mg] (mean ± SD)	p110α/PTEN dKO eye mass [mg] (mean ± SD)	P value (two sample t-test)
1 week	8.9 ± 1.9	9.8 ± 0.7	0.040
2 weeks	13.4 ± 0.5	13.9 ± 0.5	0.059
5 weeks	18.1 ± 1.1	18.9 ± 1.5	0.025
8 weeks	21.1 ± 1.2	22.9 ± 2.0	0.003
12 weeks	22.1 ± 1.5	22.2 ± 2.2	0.473
